# Early Warning of Systemic Risk in Commodity Markets Based on Transfer Entropy Networks: Evidence from China

**DOI:** 10.3390/e26070549

**Published:** 2024-06-27

**Authors:** Yiran Zhao, Xiangyun Gao, Hongyu Wei, Xiaotian Sun, Sufang An

**Affiliations:** 1 School of Economics and Management, China University of Geosciences, Beijing 100083, China; yiran_zhao0815@163.com (Y.Z.); weihongyu2018@126.com (H.W.); urnotsun@163.com (X.S.); 2 Key Laboratory of Carrying Capacity Assessment for Resource and Environment, Ministry of Natural Resources, Beijing 100083, China; 3 School of Management, Hebei GEO University, Shijiazhuang 050031, China

**Keywords:** transfer entropy, causality networks, risk contagion, early warning, commodity markets

## Abstract

This study aims to employ a causal network model based on transfer entropy for the early warning of systemic risk in commodity markets. We analyzed the dynamic causal relationships of prices for 25 commodities related to China (including futures and spot prices of energy, industrial metals, precious metals, and agricultural products), validating the effect of the causal network structure among commodity markets on systemic risk. Our research results identified commodities and categories playing significant roles, revealing that industry and precious metal markets possess stronger market information transmission capabilities, with price fluctuations impacting a broader range and with greater force on other commodity markets. Under the influence of different types of crisis events, such as economic crises and the Russia–Ukraine conflict, the causal network structure among commodity markets exhibited distinct characteristics. The results of the effect of external shocks to the causal network structure of commodity markets on the entropy of systemic risk suggest that network structure indicators can warn of systemic risk. This article can assist investors and policymakers in managing systemic risk to avoid unexpected losses.

## 1. Introduction

The commodities market has been rapidly growing in recent years, with an increase in the number of investors and a spike in the volume and value of transactions [1,2]. The importance of the global economy and business activities is increasing. The financialization of commodity markets has led to increased correlations among different commodities [3], and the role of interactions between spot and futures markets is evident [4]. In addition, extreme events such as COVID-19 and Russia–Ukraine geopolitical shocks caused major changes in the supply situation of key supplying countries [5], disrupting the original supply–demand balance and causing sharp fluctuations in the prices of some commodities [6]. Concurrently, this has engendered apprehensions among market investors regarding commodity markets, influencing investment portfolios, and hedging strategies pertaining to commodities [7]. Extreme event shocks can cause cascading effects among commodity markets [8], triggering systemic risk [9]. Once systemic risk arises, it may not only lead to losses in the commodity market but also pose a serious challenge to the management of systemic risk in the commodity market. Therefore, early warning of systemic risk in commodity markets is crucial.

Systemic risk mainly emphasizes avoiding the risk of disrupting financial services due to simultaneous damage to all or part of the financial system [10], which could have severe negative effects on the real economy and the entire financial market [11]. Exploring the influencing factors of systemic risk and providing early warning information can provide new solution ideas to mitigate risk shocks in advance [12]. Previous studies have examined the influences of systemic risk from two main perspectives. On the one hand, studies have emphasized the micro characteristics of financial institutions, such as size, capital, and governance factors [13]. On the other hand, it has been emphasized that the cause is the interconnected risk among different market entities [14], with interactions between various commodities and markets amplifying localized negative impacts, potentially triggering systemic risk [15]. Commodity markets are highly correlated in terms of price fluctuations because of the existence of linkages in processing or the influence of the external environment and policy regulation [16], and a resonance effect is observed [17]. The spillover relationship based on the GARCH-BEKK model and the cross-correlations based on DCCA can help identify and quantify the interrelations among financial markets across different time scales [18,19]. The results show that the outbreak of financial crises leads to changes in the linkages among financial markets [20,21]. This highlights the importance of focusing on the dynamic relationships among commodity markets [22].

In related research, the interconnections among the energy, industrial metals, precious metals, and agricultural commodity markets have become a hot issue for scholars [23,24,25]. On one hand, as energy commodity prices rise, the demand for biofuels as alternative energy increases, driving up the prices of agricultural products like corn and soybean oil, which are used as biofuel raw materials [26,27]. On the other hand, rising energy prices increase the transportation and production costs of agricultural products and industrial metals [20], thereby pushing up their commodity prices [28]. Additionally, higher energy prices enhance the appeal of precious metals as safe-haven and hedging instruments, thus affecting precious metals commodity prices [29]. Therefore, energy commodity prices play a significant role in influencing the prices of various commodity markets [30,31]. Additionally, because of rapid economic growth, China has become one of the world’s largest consumers of commodities [32], making the dynamics of the Chinese commodity market a subject of great interest [33,34,35]. This has motivated us to focus on commodity markets related to China and to further explore the internal operating mechanisms and dynamic changes in different categories of commodity markets.

The price time series of commodities typically exhibit nonlinear characteristics [36]. Distinct from prior research that directly employed Granger causality to measure the degree of interconnectedness within the financial system [37,38], we conduct a more in-depth investigation into the nonlinear causal dynamics among commodity prices. Transfer entropy (TE) naturally combines the linear and nonlinear information flow of time series [39,40] and does not assume any specific model for the causal effect existing between two systems, which is superior for revealing the causal relationship between two time series [41]. The causality method based on TE can effectively measure the causal relationships in commodity price time series with nonlinear characteristics [42], and it can effectively distinguish between driving factors and responding factors. It has a wide range of applications in many fields such as biology, climatology, brain science [43], and finance [44]. Previous studies have used TE to analyze information transfer among different categories of commodity markets. For example, it has been used to quantify information transfer among clean energy, carbon, oil, and precious metals [45] and to study the information flow paths between Brazilian ethanol and sugar prices and global oil prices [46]. TE has also been utilized to investigate the characteristics of information transfer networks for the prices of international commodities in the energy, metal, and agricultural sectors [47]. Additionally, studies have explored the information transfer between commodity markets and stock markets, including the Chinese stock market and African stock markets [39,48].

Therefore, this study selects futures and spot prices of 25 commodities related to China, from four types including energy, industrial metals, precious metals, and agricultural products, as the sample data. First, we investigate the nonlinear causal relationships among commodity prices from the perspective of network dynamic evolution, identifying the commodity markets that play an important role. Second, the dynamic evolution process of causal relationships among commodity markets is examined, uncovering the characteristics of the evolution of commodity market causal relationship structures under various extreme events. Finally, we measure the impact of external shocks on the systemic risk entropy through the commodity market causal relationship network structure. The findings suggest that the characteristics of the causal network structure among commodity markets can serve as early warnings for systemic risk. This article can assist investors and policymakers in managing systemic risk to avoid unexpected losses.

## 2. Materials and Methods

### 2.1. Data and Sample

According to the NASDAQ Commodity Family Report, commodity markets primarily consist of three categories including energy, metals (including industrial and precious metals), and agricultural products [49]. Our sample covers different fields. Additionally, our study considered market diversity, including the Chinese commodity trading market, the CME Group market, and the London Metal Exchange (LME) market. The futures and spot trading in these markets account for a significant share of global commodity trading. Therefore, we selected a total of 25 commodities as the research sample for this study [23,24,25]. The data cover the period from 22 February 2010 to 4 March 2022, for a total of 2528 daily futures or spot prices in each time series.

Table 1 shows a detailed description of the data set. Furthermore, a larger number of commodities was considered including energy (natural gas, crude oil, heating oil), industrial metals (copper, nickel, cobalt, aluminum, tin, zinc), precious metals (gold, silver), and agricultural products (canola oil, soybeans, corn, wheat, soybean oil, strong wheat). All the data were obtained from the websites of the Energy Information Administration (http://www.eia.gov, accessed on 12 March 2022), https://www.investing.com (accessed on 12 March 2022), and the wind database.

In order to facilitate analysis, the data were uniformly logarithmic processed in this paper, and the specific formula is as follows:

(1) $$L_{i,t}=\ln\left(\frac{Q_{i,t}}{Q_{i,t-1}}\right)$$

where $L_{i,t}$ represents the logarithmic return of the ith variable at moment *t*, and $Q_{i,t}$ represents the original price of the *i*-th variable at moment *t*. This study is based on the obtained log-return series for analysis.

### 2.2. Transfer Entropy Networks of Commodities

The transfer entropy (TE) method was proposed by T. Schreiber based on information entropy [50]. This method analyzes the information transfer among variables by examining whether a past state of a variable may reduce the uncertainty in the future state of another variable. The TE method can measure the imbalanced causal relationship between two time series, and, more importantly, it is a powerful technique for quantifying the coupling strength and asymmetric properties of dynamic systems. In this paper, we use transfer entropy to quantify the causal relationship among commodities prices. Next, we briefly describe the calculation of transfer entropy. First, according to Shannon’s information theory [51], the minimum amount of information required to optimally encode an independent discrete variable, X, that follows a probability distribution is:

(2) $$H_{X}=-\sum_{i}p\left(i\right)\log_{2}p\left(i\right)$$

The units of Shannon entropy are bits. Thus, if we use a different probability distribution q(i) instead of the correct probability distribution p(i) to encode information, the extra bits in the encoding process can be calculated by Kullback entropy, also called relative entropy [52]:

(3) $$K_{X}=\sum_{i}p\left(i\right)\log\frac{p\left(i\right)}{q(i)}$$

In addition, we must consider the probability of the transition state. In the k-order stationary Markov process, if we know all the previous states, the average bits required to encode a new state are:

(4) $$h_{X}=-\sum p\left(x_{n+1},x_{n},\cdots ,x_{n-k+1}\right)\log p\left(\left.x_{n+1}\right|x_{n},\cdots ,x_{n-k+1}\right)$$

Since the above equation satisfies the Markov property, it is equivalent to the difference in Shannon entropy with delays of k+1 and k, $h_{X}=H_{X^{\left(k+1\right)}}-H_{X^{\left(k\right)}}$.

For the transfer entropy of joint probability, we need to consider two series, X and Y, with lags of order, k and l, respectively. If there is no information transfer from X to Y, adding the state of X does not affect the state of Y. Then, the following generalized Markov property hypothesis holds:

(5) $$p\left(\left.y_{n+1}\right|y_{n_{j}}\ldots ,y_{n-l+1}\right)=p\left(\left.y_{n+1}\right|y_{n},\ldots ,y_{n-l+1},x_{n},\ldots ,x_{n-k+1}\right)$$

Otherwise, if the state of adding X exerts an impact on Y, then information flows from X to Y. This deviation can be quantified by Kullback entropy, according to which the transfer entropy is defined.

(6) $$T_{X\to Y}=\sum p\left(y_{n+1},y_{n}^{\left(l\right)},x_{n}^{\left(k\right)}\right)\log\frac{p\left(\left.y_{n+1}\right|y_{n}^{\left(l\right)},X_{n}^{\left(k\right)}\right)}{p\left(\left.y_{n+1}\right|y_{n}^{\left(l\right)}\right)}$$

Based on the Markov property of financial time series, k=1 and l=1 in this paper [53]. In the causality inference, we count the negative entropy value as 0 in the calculation because it has no positive effect.

**Figure 1:**
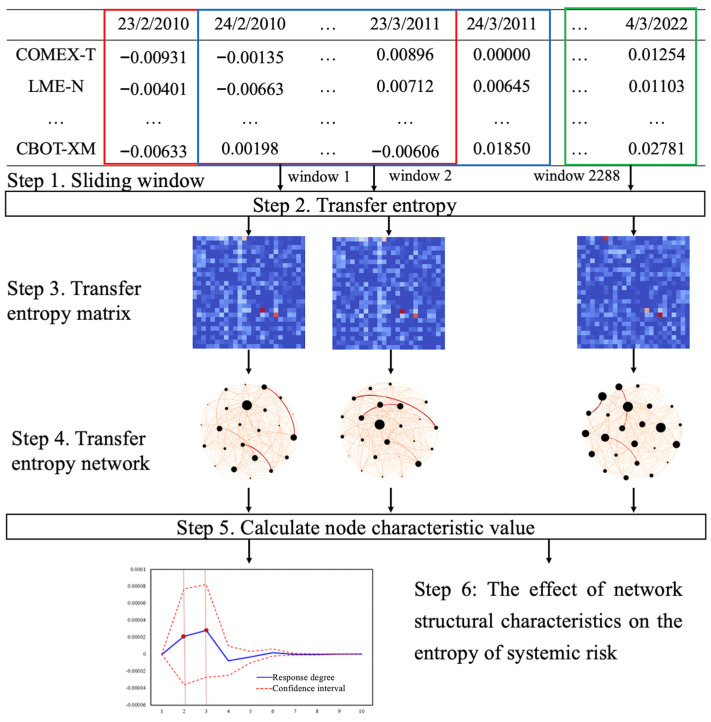
The overarching framework of the study method.

In order to better identify the evolution law of multi-dimensional commodity market prices over time and the causal relationship among commodities, the construction process of the causal relationship network is described as follows, as shown in Figure 1. **(1)** Set the sliding window to obtain the dynamic series of commodity evolution over time. **(2)** Combine the sliding window method to calculate the transfer entropy among the prices of individual commodities under each window. **(3)** Constitute the calculated transfer entropy into a matrix and represent the transfer entropy matrix as shown in Equation (7). **(4)** Construct a directed weighted complex network based on the transfer entropy matrix. Generally, a directed weighted complex network ${Net}_{m}=(N,L,W)$ includes a set of nodes $N=\left\{n_{1},\;n_{2},\ldots \;n_{N}\right\}$, a set of edges $L=\left\{L_{1},\;L_{2},\ldots \;L_{N}\right\},$ and a set of weights $W=\left\{w_{1,1},\;w_{1,2},\ldots \;w_{i,j}\right\}$. The weight $w_{i,j}$ is the weight of the connecting edge from node i to node j. If there is no edge connection between node i and node j, then $w_{i,j}=0$. In this paper, we use nodes in the network to represent commodities, and the connecting weight $t_{\left(x,y\right)}$ of node X and node Y represents the transfer entropy between the two nodes. If $t_{\left(x,y\right)}=0$, node X and node y have no edge. Therefore, the causality networks of commodity prices based on transfer entropy under different windows are constructed, and then the causality models among commodities are analyzed. **(5)** Calculate the characteristic value and average values of each node in the directed weighted complex network under each window. In this paper, the network characteristic values such as out-degree, out-strength, and weighted betweenness centrality are selected to characterize the network topology structure. **(6)** Identify and analyze the network topology meaning of the eigenvalues obtained, so as to identify the causal relationship among commodity prices.

(7) $$TE=\left[\begin{array}{ccc} \begin{array}{cc} \begin{array}{c} t_{1,1}\\ t_{2,1} \end{array} & \begin{array}{c} t_{1,2}\\ t_{2,2} \end{array} \end{array} & \cdots & \begin{array}{c} \begin{array}{cc} t_{1,24} & t_{1,25} \end{array}\\ \begin{array}{cc} t_{2,24} & t_{2,25} \end{array} \end{array}\\ \vdots & \ddots & \vdots \\ \begin{array}{cc} \begin{array}{c} t_{24,1}\\ t_{25,1} \end{array} & \begin{array}{c} t_{24,2}\\ t_{25,2} \end{array} \end{array} & \cdots & \begin{array}{cc} \begin{array}{c} t_{24,24}\\ t_{25,24} \end{array} & \begin{array}{c} t_{24,24}\\ t_{25,25} \end{array} \end{array} \end{array}\right]$$

In the above matrix, $t_{\left(x,y\right)}$ is the transfer entropy of the *x*-th group of time series to the *y*-th group of time series. When x=y, $t_{\left(x,y\right)}=0$.

### 2.3. Measures of Systemic Risk Entropy and Network Structure

#### 2.3.1. Systemic Risk Entropy

In this paper, we use the systemic risk entropy indicator to measure the overall risk of the commodity price system in order to help identify and characterize the overall level of risk in the system. The indicator is mainly constructed based on the eigenvalues of the correlation matrix, which can reflect the information of the real market. The specific definitions are as follows [54].

(8) $${RE}_{H}=\left(-\frac{1}{log\left(N\right)}\right)\times \sum_{i=1}^{H}\left(\frac{\lambda_{i}}{N}\right)\times \log\left(\frac{\lambda_{i}}{N}\right)$$

where $\lambda_{i}$ represents the characteristic value that can reflect the real market information, H is the number of characteristic values, and N is the number of variables. Generally speaking, ${0\leq RE}_{H}\leq 1$. The closer the value of systemic risk entropy is to 0, the higher the overall risk of the system.

#### 2.3.2. Measures of Network Structure of Commodities

(1) Degree

Degree is the number of nodes that have direct contact with a particular node in a causal relationships network of commodities [55]. The greater the degree of a node, the greater number of one commodity is correlated with other commodity price movements. Out-degree is the number of influence that commodity markets have on other commodities, and in-degree is the number of one commodity direct received from others. The out-degree and in-degree are calculated as follows:

(9) $$K_{i}^{out}(t)=\sum_{j=1}^{n}a_{ij}(t)$$

(10) $$K_{i}^{in\;\;\;}(t)=\sum_{j=1}^{n}a_{ji}(t)$$

where if commodity i has a causal relationship with commodity j during sliding window t, an edge from i to j is drawn, and $a_{ij}\left(t\right)$ is the adjacent matrix of the network. Otherwise, no edge is drawn, and $a_{ij}\left(t\right)=0$. The out-degree $K_{i}^{out}\left(t\right)$ of country i in window t is $a_{ij}\left(t\right)$, and the in-degree $K_{i}^{in}\left(t\right)$ of country i in window  t  is $a_{ji}\left(t\right)$.

(2) Strength

The strength level of the causal relationship among commodity price fluctuations can be reflected by strength. A commodity with a higher strength value has a higher causal relationship with other commodity markets in price fluctuations. The strength of one node denotes the total weights of the links connected to it, and in a directed network, strength includes in-strength and out-strength. In this paper, in-strength reflects the total causal relationship that one commodity obtains from other commodities’ price fluctuations, and out-strength reflects the total causal relationship that one commodity affects other commodities’ price fluctuations. The in-strength and out-strength are calculated as follows:

(11) $$S_{i}^{out}\left(t\right)=\sum_{j=1}^{n}a_{ij}\left(t\right)*w_{ij}(t)$$

(12) $$S_{i}^{in}(t)=\sum_{j=1}^{n}a_{ji}\left(t\right)*w_{ji}(t)$$

where $w_{ij}\left(t\right)$ is the weight of the edge from i to j in sliding window t, which is the extent of the causal relationship when commodity i exports to commodity j during window t.

(3) Betweenness centrality

Betweenness centrality measures how much a node is in the “middle” of the other node pairs in the network. If many couples need to meet each other through a matchmaker, then this matchmaker is very important. The larger the value is, the stronger the mediation of the node. The betweenness of one node is defined by the number of shortest paths going through it. Betweenness centrality usually measures the mediation capability of nodes in the network and is calculated based on the distance and mainly used to measure the topology of the structure. Betweenness centrality is calculated as follows [56]:

(13) $$B_{i}=\frac{\sum_{j}^{n}\sum_{k}^{n}g_{jk}\left(i\right)∕g_{jk}}{\left(n-1\right)\left(n-2\right)},j\;\neq \;k\;\neq \;i,\;j<k$$

where $g_{jk}$ is the total number of the shortest paths between nodes j and k. $g_{jk}(i)$ is the number of shortest paths between nodes j and k that pass through node i. n is the number of commodities in the network.

### 2.4. Early Warning of Commodity Market Risks

Impulse response functions can provide a suitable model to quantify the impact of shocks from external unexpected events. In this paper, we analyze the degree of response of shocks to changes in the structure of causal networks to systemic risk under consideration of the impact of external event shocks by constructing an impulse response function based on a VAR model. In this paper, the network topology indicators obtained based on the causal network model and the systemic risk entropy are used as endogenous variables to construct a multidimensional vector autoregressive (VAR) model. At this point, the VAR model of N-element P-order can be expressed as follows.

(14) $$\;\;\;\;\;Y_{t}=\alpha +\sum_{i=1}^{p}{\beta_{i}Y}_{t-i}+\varepsilon_{t}t=1,2,\ldots ,p\;\;$$

where $Y_{t}$ is a k-dimensional column vector of endogenous variables that represents the entropy of the systemic risk under a sliding window. *P* is the lag order and t are the number of samples. $\beta_{i}$ is a k × k dimensional matrix, which is the matrix of coefficients to be estimated. $\varepsilon_{t}$ is a k-dimensional column vector of perturbations that can be correlated contemporaneously with each other but not with their own lagged values and not with the variables on the right-hand side of the equation.

An impulse response function is constructed based on the VAR model to measure the degree of impact on the systemic entropy of risk in the current and future values given a one-unit positive shock to the random error term. It can reflect the influence degree of systemic risk entropy when the network structure is affected by external shocks in different situations. The impulse response function is shown below, where L is the lag factor, $I_{k}$ is a k-dimensional fixed matrix, and the i-th variable in $y_{t}$ is $y_{it}$, which can be expressed as follows.

(15) $$\;\;\;y_{it}=\sum_{j=1}^{k}(a_{ij}^{\left(0\right)}e_{jt}+a_{ij}^{\left(1\right)}e_{jt-1}+a_{ij}^{\left(2\right)}e_{jt-2}+\ldots ),\;t=1,2,\ldots T\;$$

Suppose we give a standard deviation shock to $y_{1}$ at moment *t* = 0.

(16) $$\;\;e_{1t}=\left\{\begin{array}{c} 1,\;t=0\\ 0,t=1,2,3,\ldots \end{array}\right.,\;e_{it}=0,t=0,1,2,\ldots i\neq 1$$

Therefore, when $y_{1}$ is subjected to an external shock, the response of $y_{j}$ is $a_{1j}^{\left(0\right)},a_{1j}^{\left(1\right)}$, …; the formula is as follows.

(17) $$a_{ij}^{\left(q\right)}=\frac{\partial y_{i,t+q}}{\partial e_{jt}},q=0,1,2,\ldots ,N,\;t=1,\;2,\;\ldots ,T$$

## 3. Results

### 3.1. Subsection Structure of Causal Relationship Network

**Figure 2:**
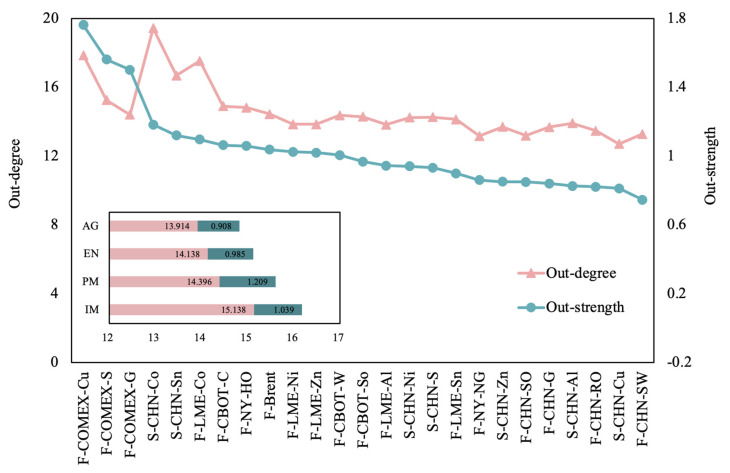
The average out-degree of commodities in causal networks.

Transfer Entropy Causal Networks (TECNs) are superior in revealing deep interactions and causal mechanisms. They emphasize the directionality of the information flow, which can help us to identify the key “sources” of information within the system. The out-degree of a TECN is used to measure the influence number of commodity markets. The dynamic out-degree results can be used to capture how and how much information about price fluctuations is transmitted from the commodity market to other markets at each moment in a time period. On this basis, the average out-degree is calculated to identify commodity markets as the main disseminators of information over the study interval. The results in Figure 2 show that about 20% of commodity markets act as disseminators of information on price fluctuations with an out-degree of more than 15. Overall, the average out-degree of the commodity causality network is 14.61, which means that the price fluctuations in commodities have a direct impact on the price fluctuations in more than half of the other commodities. The network connectivity among commodity markets related to China is high, with an obvious effect on the transmission of information. This paper complements the results of previous scholars’ identifications of key players in commodity markets based on spillovers by finding that commodities with high outliers belong to industrial and precious metals [33], including Chinese cobalt spot, COMEX copper futures, LME cobalt futures, Chinese tin spot, and COMEX silver futures. China’s rapid industrialization has resulted in a significant increase in demand for industrial metals. The metals market plays a prominent role in the real economy, with China being the largest consumer of refined copper and cobalt. The importance of China’s commodity market is also growing in the financial market sector [57]. As previous findings have shown that industrial metals are highly correlated with other commodities [58], copper has been shown to be crucial in commodity price information transmission [59]. The results of this paper further identify the commodity categories that are highly influential in different markets. The price volatility of mineral resources has a wider impact relative to agricultural and energy products. Mineral resources have a wide range of applications, and, at the same time, because of the rise in clean energy, the importance of mineral resources such as cobalt and copper has been elevated to a strategic level. But these curial metals are affected by geopolitics and resource endowment, and the magnitude of changes in their supply and demand is large, resulting in greater volatility in the market price and a wider scope of contagion in the market price.

The out-strength measures the degree of ability of each commodity to influence the network. The average out-strength for commodities is 1.03. The volatility of the two curves in Figure 2 is not synchronized, which implies that the scope and intensity of the influence of commodities are not completely synchronized. The out-strength of COMEX copper futures, COMEX silver futures, and COMEX gold futures are much larger than those of the other commodities in the network. The results identify that NYMEX financial products are relatively more influential, and their price fluctuations can easily cause other commodities’ price movements. In addition, the volatility of gold and silver, which are preferred as risk hedges, reflects to some extent the level of investor panic and therefore has a greater impact on other futures markets as well. Considering the out-strength and out-degree together, we find that the greater influence and scope of influence belongs to precious metals and industrial metals, while the degree of influence and influence of agricultural products are relatively small, which may be more related to the rapid development of industry and economy in recent years.

**Figure 3:**
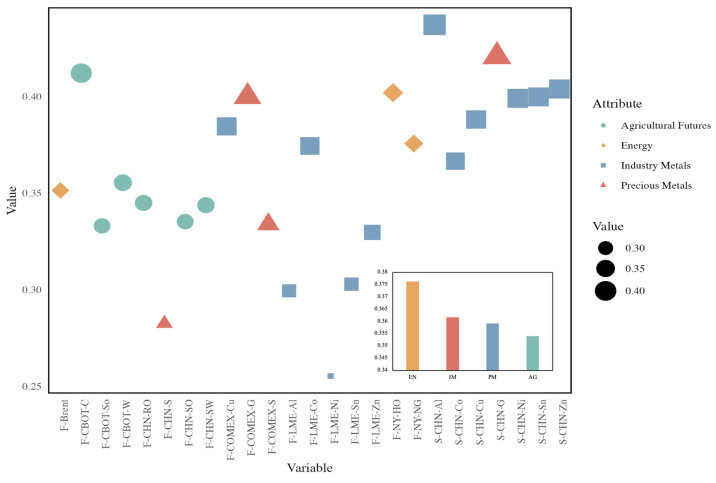
The average out-strength of commodities in causal networks.

To measure the ability of each commodity to act as a bridge in the network, we use standardized betweenness centrality to identify the bridging capacity of commodity prices. Figure 3 shows the average betweenness centrality of all commodities. The average of the network is 0.36. The strongest bridge capability in the network is Chinese aluminum spot, which indicates that Chinese aluminum spot transmits more information in the network. Three of the four products with the weakest transmission capacity are from the LME market, including LME nickel futures, LME aluminum futures, and LME zinc futures. This suggests that the LME’s product “bridges” are relatively weak and rarely act as intermediaries to transmit information about price fluctuations in the network, which can have an impact on the prices of other commodity markets. According to the classification of each product’s statistics, we found that energy commodities are the most important bridge in the network, which is consistent with the conclusions of previous studies [31]. Energy is the basis for industrial development and an indispensable resource. Whether it is traditional industrial manufacturing or agriculture, it cannot be separated from the support of energy products, and its price fluctuations are more likely to undertake a variety of commodity price changes. The impact of its price volatility is more likely to take on a variety of commodity price changes.

**Figure 4:**
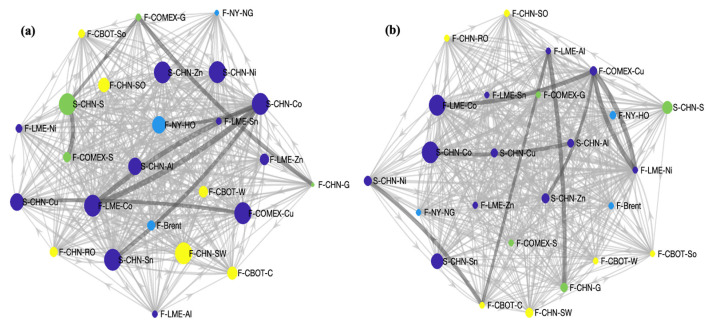
The causal relationship network of commodities. (**a**) The causal relationship network sorted by in-degree; (**b**) The causal relationship network sorted by in-strength. Note: Each node represents a different commodity market, and each side represents the causal relationship between the commodity markets. The greater the weight of the edge, the deeper the color. The color of the nodes represents the following: blue, energy; purple, industrial metals; green, precious metals; yellow, agricultural products.

The transfer entropy causality network is shown in Figure 4. The node size in Figure 4a is arranged according to in-degree. Markets with a high number of exposures to other commodities fall mainly into the category of industrial metals and precious metals products. The leading commodities are COMEX copper futures, LME cobalt futures, and Chinese nickel, cobalt, tin, zinc, and silver spot. The main markets that are affected by other commodities in small quantities are Chinese gold futures, NYMEX natural gas futures, and COMEX gold futures. Figure 4b shows the results of the nodes arranged according to in-strength, and the markets with a higher intensity of influence by other commodity prices still belong mainly to industrial and precious metals. In order, these are Chinese cobalt spot, LME cobalt futures, Chinese tin spot, and Chinese gold spot. Markets with a lower intensity of influence from other commodity market prices are, in order, LME tin futures, CBOT soybeans, COMEX gold, CBOT corn, and NYMEX natural gas futures.

**Figure 5:**
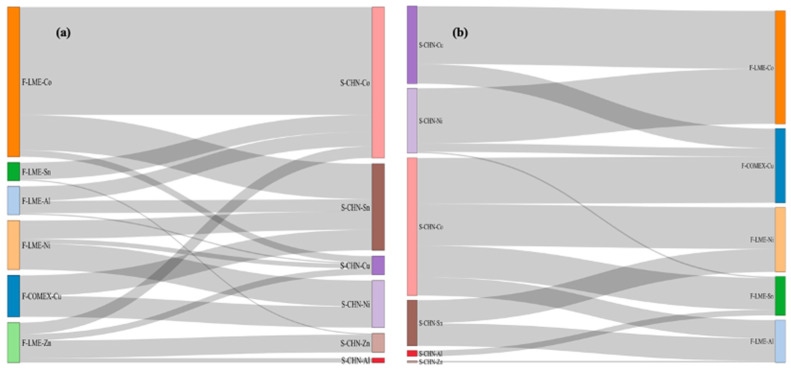
The causal relationship network of commodities. Note: The Sankey diagram illustrates the transfer entropy between metal futures and spot markets. The width of the flows represents the magnitude of the transfer entropy; the wider the flow, the higher the transfer entropy. Each market is represented by a different color. Panel (**a**) shows the transfer entropy from metal futures markets to Chinese metal spot markets, and panel (**b**) shows the transfer entropy from Chinese metal spot markets to futures markets.

The importance of industrial metals in the commodity market deserves attention. Therefore, further analysis is conducted by taking the industrial metals market as an example. Figure 5 intuitively illustrates the transfer entropy between metal futures and spot markets. The flow width represents the size of the transfer entropy, with a larger width indicating higher transfer entropy. Each market is represented by a different color. Figure 5a shows the transfer entropy from the metal futures market to the Chinese metal spot markets. The highest transfer entropy is from LME cobalt futures to Chinese cobalt and tin spot markets, followed by LME nickel futures to the Chinese nickel spot market. Overall, LME cobalt futures have a strong correlation with the Chinese metal spot market.

Figure 5b shows the transfer entropy from the Chinese metal spot market to the futures market, with the highest transfer entropy from Chinese copper and nickel spot markets to LME cobalt futures. Overall, the Chinese cobalt spot market has a strong correlation with the metal futures market. These results indicate a strong causal relationship between the futures and spot markets for these commodities, consistent with previous research findings that futures and spot markets have a price discovery function and exhibit price correlations [60]. The price correlations among different metal commodities are also significant, as the connections in the supply chain and application industries result in strong causal relationships in commodity market prices. The results of the analyses provide a scientific basis for commodity markets to identify the sources of contagion of price volatility and inform the identification of systemically important commodities and the management of systemic risk in commodity markets.

### 3.2. Time-Varying Analysis of the Causality Network

**Figure 6:**
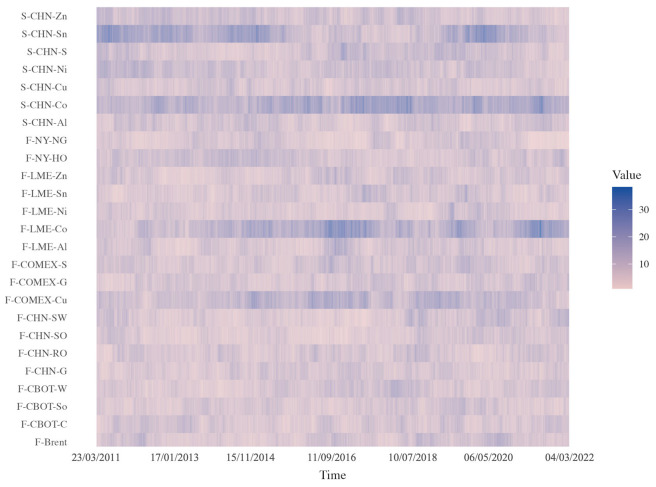
The evolution of betweenness centrality over time. Note: The colors represent the degree of betweenness centrality. Darker colors indicate a stronger role of the commodity as a bridge in transmitting information to other commodity markets. The color gradient shows changes in its intermediary role over different periods.

In order to better identify the time-varying characteristics of the causal relationship network of commodities, the trend in the intermediate centrality and output of these commodities with a sliding window was expressed. The results of betweenness centrality are shown in Figure 6. On the whole, the time period with strong betweenness centrality is continuous within a period of time, indicating that the causal network has local stability and overall time variability.

From 2010 to 2011, Chinese tin spot had an obvious betweenness centrality. This period was in the late stage of the financial crisis and the gradual recovery of the global economy. The increase in actual demand stimulated the rise in the global tin price, and tin products provided an intermediary bridging function as the market information transmission of other products. The cobalt futures and spot markets showed a high degree of intermediary centrality over the period 2013–2022, with demand for cobalt increasing as a result of the boom in electric vehicles. However, the relatively limited availability of high-quality cobalt mines globally and challenges in the production process, such as environmental and labor issues, have led to supply constraints. As a result, during periods of supply–demand imbalance, investors often have optimistic or pessimistic expectations and trade to drive prices up or down, and their high price volatility makes them a strong intermediary of market information.

**Figure 7:**
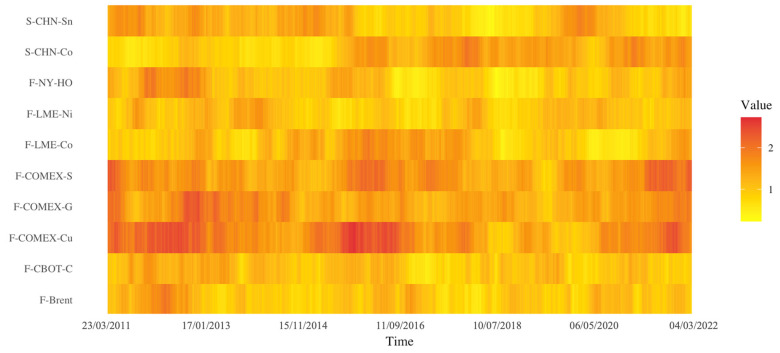
The evolution of out-strength over time. Note: The colors represent the size of the average out-strength value. Darker colors indicate a greater impact of the commodity on price changes in other commodity markets. The color gradient shows changes in its influence over different stages.

The results of the average out-strength of the top ten commodities are shown in Figure 7, i.e., the extent to which commodities cause price movements in other markets. There are three obvious periods as follows: first, during the 2010–2012 European debt crisis, there was a steep increase in the out-strength of the precious metals market. This implies a larger impact on the volatility of other commodity markets, where precious metals have been considered popular hedging instruments in previous studies. Especially during the crisis, the safe-haven function of precious metals rose rapidly, and investor confidence declined [61]. This is more likely to cause massive price volatility in other financial markets.

Second, around 2015, the impact of copper futures on other commodity markets strengthened. On the one hand, crude oil prices experienced a significant decline during this period, and the OPEC production cut agreement overlaid with the increase in shale oil production in the U.S. led to heightened concerns about crude oil oversupply in the market. At the same time, there was a slowdown in the metals market, with metals such as copper facing weak demand. Global economic factors and supply and demand relationships, among others, influence the interplay of prices in the commodities market. In addition, the impact of precious and industrial metals on other commodity markets in 2020–2022 was significantly higher. With the impact of the epidemic superimposed on the outbreak of the Russian–Ukrainian conflict, Russia suddenly reduced a large number of mineral exports, resulting in the commodities of industrial metals price changes. The impact also spread to other areas such as energy, agricultural products, etc. At the same time, under the crisis, investors were not optimistic about expectations, so the precious metals market heat increased. The market information changes are more likely to be transmitted to other commodity markets, resulting in large-scale risk contagion.

### 3.3. Causal Model External Shock and Early Warning Analysis

**Figure 8:**
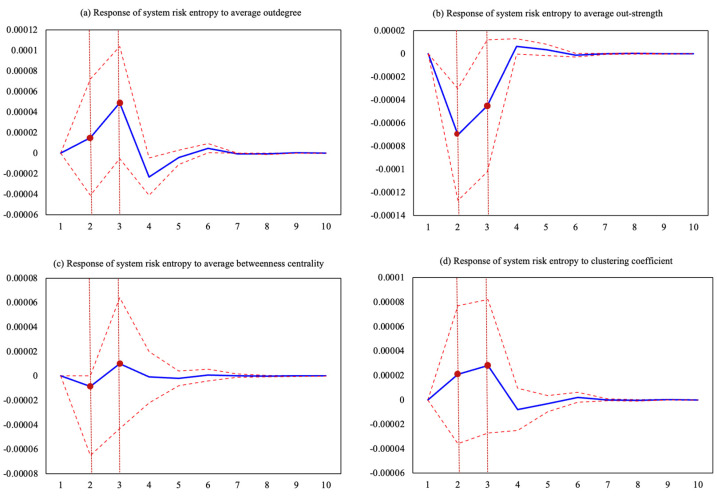
Impulsive effects of causal network structure on systemic risk entropy. (**a**) Response of system risk entropy to average outdegree; (**b**) Response of system risk entropy to average out-strength; (**c**) Response of system risk entropy to average betweenness centrality; (**d**) Response of system risk entropy to clustering coefficient. Note: The blue line represents the corresponding degree; the red line represents the confidence interval.

The corresponding changes in system risk entropy when the price causality network structure is impacted are shown in Figure 8. The horizontal axis represents the impact time, and the vertical axis represents the response degree of system risk entropy. In this model, some variables are removed because they have obvious multicollinearity. It can be seen that the influence of one-unit average out-degree on system risk entropy showed an upward trend in the first two periods, reached the maximum value of 0.0049% in the third period, and then this influence gradually subsided. The impact of the average out-strength shock on the system risk entropy declined in the first two periods and reached a trough in the second period, with a decrease of about −0.007%. In the third period, the system risk entropy showed an upward trend and a positive effect but gradually stabilized. In comparison, the influence of the network’s average betweenness centrality on the system risk entropy reached its maximum value in the third phase, with an impact of 0.001%. The influence of the network’s overall clustering coefficient on the system risk entropy showed a rising trend in the first two phases and reached its maximum value of 0.0028% in the third phase. The influence of system risk entropy on the system itself is the largest in the first lag period, with an impact of 0.1393%. However, this influence decreased rapidly as time passed. The causal network structure of commodity markets, when impacted by extreme events, has a significant effect on the systemic risk of commodity markets. The results indicate that the impact is particularly strong within three months of the extreme event. During this phase, attention should be paid to market anomalies, especially for commodities that play a pivotal role in the market. The analytical results provide a quantitative basis for systemic risk early warning.

Table 2 demonstrates the regression results of the TECN structural indicators of the commodity market on the systemic risk entropy. Out-strength, out-degree, and betweenness centrality all have a significant effect on systemic risk entropy, with *p*-values less than 0.01. The coefficient of influence of out-strength on the entropy of systemic risk is 0.02, and the coefficients of influence of out-degree and betweenness centrality are −0.002 and −0.055, respectively. This suggests that the relationship of price volatility contagion among commodities affects the systemic level of risk in the market and that the observation of the relationship among commodities can be used to provide further early warning of market risk.

## 4. Conclusions and Discussion

This paper constructs a dynamic causal network using transfer entropy and complex network analysis methods. Firstly, the results of the causal relationship network structure among commodity markets indicate that COMEX copper futures, as the sender of information transmission, have the highest impact on other commodity markets. Chinese cobalt spot, as the receiver of information transmission, is most affected by other commodity markets. Chinese aluminum spot, as a network bridge, has a significant influence on information transmission. Secondly, the evolution of the causal relationship network in commodity markets exhibits evident time-varying characteristics. During the European debt crisis (2010–2012), the COVID-19 pandemic, and the Russia–Ukraine conflict (2020–2022), the network structure characteristics changed significantly, with industrial and precious metals markets exerting stronger price influences on other commodity markets. Lastly, using the VAR-based impulse response function, we found that changes in the causal relationship network structure, impacted by external events, significantly affect systemic risk entropy, typically influencing outcomes with a lag of two to three periods. Observing changes in the network structure can effectively predict systemic risk variations.

Therefore, our findings suggest that investors and policymakers need to be prudent in investing and managing commodities. Paying attention to key commodities in the market, such as industrial and precious metals, is crucial for informed decision-making. When formulating portfolio strategies and risk management measures, it is important to focus on the price information transmission among different commodity markets. Given the time-varying nature of information transmission, investors should consider continuously monitoring local geopolitical situations and policy changes, as these extreme events may significantly impact the internal structure of commodity markets. This would enable them to consider different commodity investment allocation schemes over various time frames. Finally, the causal relationship network structure of the commodity markets serves as an early warning mechanism for systemic risk. Thus, investors should not only focus on specific commodity markets but also on interconnected multi-markets and multi-products. For Chinese investors and managers, it is particularly important to pay attention to the causal relationships between the Chinese commodity market and other markets to avoid greater losses due to the chain reaction affecting systemic risk.

Future research can explore the information transmission effects between more diverse international commodity markets, such as expanding the sample to include the Tokyo Commodity Exchange (TOCOM) and the Australian Securities Exchange (ASX), thereby enhancing the adaptability and applicability of this research framework. Future studies can also employ machine learning algorithms and more sophisticated advanced econometric models to explore the impact differences and predictive effects of network structure on systemic risk early warning under different types of crisis events. Observing and analyzing the information transmission network structure among commodity markets can help us identify systemic risks that are difficult to measure directly.

## Figures and Tables

**Table 1:** Data descriptions and variable abbreviations.

Type	Series	Description
Energy	F-Brent	Brent oil futures
	F-NY-HO	New York Harbor No. 2 heating oil futures (USD per Gallon)
	F-NY-NG	Natural Gas Futures Contract 1 (USD per million Btu)
Industrial metals	S-CHN-Cu	China’s domestic copper spot
	F-COMEX-Cu	COMEX copper futures
	F-LME-Ni	LME nickel futures
	S-CHN-Ni	China’s domestic nickel spot
	F-LME-Co	LME cobalt futures
	S-CHN-Co	China’s domestic cobalt spot
	F-LME-Al	LME aluminum futures
	S-CHN-Al	China’s domestic aluminum spot
	F-LME-Sn	LME tin futures
	S-CHN-Sn	China’s domestic tin spot
	F-LME-Zn	LME zinc futures
	S-CHN-Zn	China’s domestic zinc spot
Precious metals	F-COMEX-G	COMEX gold futures
	F-CHN-G	China’s domestic gold futures
	F-COMEX-S	COMEX silver futures
	S-CHN-S	China’s domestic silver spot
Agricultural products	F-CHN-RO	China’s domestic seed oil futures
	F-CBOT-So	CBOT soybean futures
	F-CBOT-C	CBOT corn futures
	F-CBOT-W	CBOT wheat futures
	F-CHN-SO	South China index soybean oil futures
	F-CHN-SW	South China index strong wheat futures

**Table 2:** Regression results of TECN structure on systemic risk entropy.

Variables	Model 1	Model 2	Model 3
Out-strength	0.008 ***	0.019 ***	0.020 ***
	(0.000)	(0.000)	(0.000)
Out-degree		−0.002 ***	−0.002 ***
		(0.000)	(0.000)
Betweenness centrality			−0.055 ***
			(0.000)
Observations	2288	2288	2288
R-squared	0.020	0.041	0.047
F-statistic	48.04	49.4	37.24
Prob (F)	0.000	0.000	0.000

Note: ***, **, and * denote statistical significance at the 1%, 5%, and 10% levels, respectively. *p*-values are in parentheses for regression coefficients.

## Data Availability

All the data were obtained from the websites of the Energy Information Administration (http://www.eia.gov, accessed on 12 March 2022), https://www.investing.com (accessed on 12 March 2022), and the wind database.
